# Development and functional testing of a novel in vitro delayed scratch closure assay

**DOI:** 10.1007/s00418-024-02292-y

**Published:** 2024-05-07

**Authors:** Yi Bing Aw, Sixun Chen, Aimin Yeo, John A. Dangerfield, Pamela Mok

**Affiliations:** 1 Celligenics Pte Ltd, Singapore, Singapore; 2 Bioprocessing Technology Institute, Agency for Science, Technology and Research (A*STAR), Singapore, Singapore; 3 Austrianova Singapore Pte Ltd, Singapore, Singapore

**Keywords:** Chronic wound, Wound healing assay, Secretome, Scratch closure assay, Diabetic wounds

## Abstract

As the development of chronic wound therapeutics continues to expand, the demand for advanced assay systems mimicking the inflammatory wound microenvironment in vivo increases. Currently, this is performed in animal models or in in vitro cell-based models such as cell culture scratch assays that more closely resemble acute wounds. Here, we describe for the first time a delayed scratch closure model that mimics some features of a chronic wound in vitro. Chronic wounds such as those suffered by later stage diabetic patients are characterised by degrees of slowness to heal caused by a combination of continued localised physical trauma and pro-inflammatory signalling at the wound. To recreate this in a cell-based assay, a defined physical scratch was created and stimulated by combinations of pro-inflammatory factors, namely interferon, the phorbol ester PMA, and lipopolysaccharide, to delay scratch closure. The concentrations of these factors were characterised for commonly used human keratinocyte (HaCaT) and dermal fibroblast (HDF) cell lines. These models were then tested for scratch closure responsiveness to a proprietary healing secretome derived from human Wharton’s jelly mesenchymal stem cells (MSCs) previously validated and shown to be highly effective on closure of acute wound models both in vitro and in vivo. The chronically open scratches from HaCaT cells showed closure after exposure to the MSC secretome product. We propose this delayed scratch closure model for academic and industrial researchers studying chronic wounds looking for responsiveness to drugs or biological treatments prior to testing on explanted patient material or in vivo.

## Introduction

Chronic wounds are defined as persistent injuries to the skin that deviate from the normal healing process, remaining open and unhealed typically for periods of up to 8 weeks or longer. They generally fall into one of three categories: diabetic foot ulcers (DFU), vascular ulcers and pressure ulcers (Piipponen et al. 2020), and patients suffering from obesity and/or diabetes are at especially high risk of developing them. In USA, 2.5% of the total population (~ 6.5 million) are impacted by such wounds and a large percentage of these patients are in the elderly with multiple comorbidities (Sen 2021). Recent reports estimate that almost one billion people suffer from wounds which are in the largest part chronic in nature (Garraud et al. 2017), and that the global advanced wound care market would reach $16.2 billion by 2030 (Reportlinker  2023). It is undisputed that current therapeutic interventions for chronic wounds are insufficient therefore causing considerable challenges to healthcare systems globally, affecting the well-being of millions of people and exerting a significant economic strain on society.

To develop better therapeutics for chronic wounds, it is important to understand the underlying biological mechanisms behind their persistent and non-healing pathology. Chronic wounds originate from acute wounds afflicted by inflammation and slow healing caused primarily by immune dysfunction. They develop an inflammatory microenvironment characterised by the presence of pro-inflammatory cells such as monocytes–macrophages that are unable to clear dead neutrophils and excessive inflammatory molecules such as tumour necrosis alpha (TNF-α), interferon gamma (IFNg) and interleukin-1β (IL-1β) (Krzyszczyk et al. 2018; Cañedo-Dorantes and Cañedo-Ayala 2019). There is also higher activity of certain enzymes such as matrix metalloproteinases and increased oxidative stress due to insufficient antioxidants (Bradshaw et al. 2000; Garraud et al. 2017). In addition, non-healing wounds often contain bacterial biofilms which independently stimulate further inflammation (Raziyeva et al. 2021). If one or more of these factors are present or develop over time in an acute wound, it is highly likely to turn chronic. This leads to common clinical features such as poor scabbing, oozing, build-up of dead tissue as well as lack of re-epithelialisation and angiogenesis (Larouche et al. 2018).

Various in vitro models have been developed to study the cellular and molecular processes involved in wound healing. However, they lack the ability to recreate the intricate characteristics that exist in patients. This shows a clear need for an in vitro system that is one step closer to an animal model. Conventional in vitro wound healing assays, such as the scratch assay or monolayer closure model, are valuable but limited in their ability to recreate the dynamic and complex interactions between different cell types and the inflammatory microenvironment. Moreover, these models primarily focus on acute wound healing and do not fully reflect the prolonged healing delay seen in chronic wounds (Martinotti and Ranzato 2020).

More recently, researchers have explored the use of three-dimensional (3D) tissue-engineered constructs, organoids and 3D bioprinting to generate complex skin mimics which can potentially bridge the gap between conventional in vitro assays and in vivo conditions (Hosseini et al. 2022). These advanced models have provided physiologically relevant platforms for studying wound healing processes (Tarassoli et al. 2018); (Kim et al. 2018). However, they are generally not freely accessible or easy to recreate for other researchers and tend to be more expensive and harder to conduct high throughput screening with.

To address these limitations and offer a closer approximation to in vivo conditions than has been previously available from other scratch assays or monolayer closure models, we aimed to develop an in vitro cell-based assay system, using human keratinocytes and dermal fibroblasts, that emulates the inflammatory setting of a chronic wound. This manuscript describes the development of this assay and assessment of its performance using parameters that reflect the key features of chronic wounds: delayed scratch closure and stimulation of inflammation.

## Materials & methods

### Human Wharton’s jelly stem cells (hWJSC) isolation, culture and cryopreservation

Human Wharton’s jelly stem cells were isolated from umbilical cords obtained from healthy neonates after at least 36 weeks gestation. Informed consent was obtained from the parents prior to umbilical cord collection which was facilitated by Celligenics Pte Ltd − a registered tissue bank in Singapore. Umbilical cords were first washed in Hanks’ balanced salt solution (HBSS) to remove the blood. Umbilical cords were then cut into pieces which were subsequently cut open lengthwise. The Wharton’s jelly was then carefully excised from each piece and cut into small pieces. These small pieces of Wharton’s jelly were then transferred into a tissue culture dish containing hWJSC culture medium (Celligenics proprietary formulation). The explanted tissue was incubated in a 37 °C incubator with 5% carbon dioxide (CO_2_). Culture medium was changed every 2–3 days while cells migrated out from the tissue. When there was substantial outgrowth from the explants, explants were removed and the cells were dissociated from the dish by incubating with TrypLE (recombinant trypsin) at 37 °C in an incubator with 5% CO_2_ for 15 min after washing with HBSS. After TrypLE incubation, the TrypLE was diluted with culture medium and the dissociated cells were collected into a centrifuge tube. Collected cells were centrifuged at 300*g* for 5 min, and resuspended in culture medium for counting after removing the supernatant. Cells were counted by trypan blue, cryopreserved in CryoStor10 and placed in liquid nitrogen for long term storage until use.

### Human WJSC characterisation

Cryopreserved hWJSCs were removed from liquid nitrogen storage and rapidly thawed in a 37 °C water bath. Thawed cells were diluted with culture medium, centrifuged at 300*g* for 5 min and resuspended in culture medium for counting by trypan blue after removing the supernatant. hWJSCs were seeded in six-well culture plates in duplicate wells. Culture medium was changed every 2–3 days, and cells were sub-cultured every 4–5 days. For trilineage differentiation, hWJSCs at passage 4 were seeded in 24-well culture plates and differentiated using StemPro™ chondrogenesis/osteogenesis differentiation kit and MesenCult™ adipogenic differentiation kit according to manufacturers’ instructions. After differentiation, cells were stained with alizarin red for osteogenesis, oil red O for adipogenesis and alcian blue for chondrogenesis, and then imaged under bright field using an inverted microscope at 4× objective.

For cell surface marker expression, passage 3 hWJSCs were dissociated by incubation with TrypLE (recombinant trypsin) at 37 °C in an incubator with 5% CO_2_ for 5 min, collected and centrifuged, and fixed in 2% paraformaldehyde (one million cells per 100 µL) at 4 °C for 15 min. After fixation, hWJSCs were washed twice by adding FACS buffer (2% FBS in PBS) to the cells, centrifuging the cell suspension and removing the supernatant for each wash. Thereafter, hWJSCs were incubated with antibodies to human CD34, CD73, CD166 (APC conjugated), CD14, CD45, CD90, and CD105 (FITC conjugated) at 4 °C for 15 min. For controls, hWJSCs were incubated with isotype (IgG) control antibodies (APC or FITC conjugated) at 4 °C for 15 min. Unbound antibody was removed by repeating the above wash step three times. The stained hWJSCs were resuspended in FACS buffer and data was acquired with FACSCelesta™ Cell Analyzer (Becton Dickinson, USA) and analysed using Facsdiva v8.0.2 (Becton Dickinson, USA).

### Secretome production

Secretome from human WJSCs was produced according to methods described in patent application WO 2023/101603 A1. Briefly, hWJSCs were seeded in Corning® HYPERFlasks®. At 95–100% confluency, culture medium was removed and hWJSCs were washed twice with HBSS. Cells were then incubated in serum-free medium. Secretome was periodically collected by removing the spent culture medium (the secretome) from the cell culture vessel and replacing with fresh medium every 2–3 days. Collected secretome was concentrated via ultrafiltration and stored at −80 °C for downstream applications.

### Scratch wound assay

Scratch wound assays were carried out in 96-well tissue culture plates using non-immortalized human adult dermal fibroblasts (HDF) and HaCaT keratinocytes. Cells were seeded in culture medium [DMEM with 5% fetal bovine serum (FBS), 1× GlutaMAX, 1× non-essential amino acid (NEAA), 1× penicillin–streptomycin] with and without an inflammatory cocktail comprising interferon gamma (IFNg) and/or phorbol 12-myristate 13-acetate (PMA) and/or lipopolysaccharide (LPS) and allowed to grow to confluence. At confluence, cells were scratched using an in-house developed scratch assay device comprising a guide template, scratch pin and imaging template (Chen et al. 2023). After scratching, the cells were washed with HBSS to remove cell debris, imaged and subsequently incubated at 37 °C in a CO_2_ incubator for 2 (HaCaT) or 3 (HDF) days in fresh culture medium with and without the inflammatory cocktail. Concentrated secretome (see above under secretome production) was added together with the final selected combination of the inflammatory cocktail for studies evaluating the effect of secretome on scratch closure in the delayed scratch closure model at the time of scratch creation. Culture medium was used as a positive control for normal scratch closure response. After scratching and before incubation, scratched cells were imaged on phase contrast at 4× objective with the imaging template to enable imaging of the same area in each well. After incubation, culture medium was removed and cells were washed with HBSS before fixing in 1% paraformaldehyde (PFA) for 30 min at room temperature. After fixation, cells were stained with 0.025% safranin O and the same area was imaged microscopically at 4× objective, on bright field using the imaging template as a guide. Scratch area pre- and post-treatment was assessed by tracing and then quantifying the area without cells in pixels using FIJI Software (Schindelin et al. 2012). The percentage wound closure was calculated by dividing the scratch area after treatment by the scratch area before treatment and multiplying this value by 100.

### Cytokine profile

HDF and HaCaT culture media after scratch under different treatment conditions were collected and stored at −80 °C until analysis. Cells treated with culture media only was used to obtain baseline cytokine levels. Cytokine levels in the culture media in the presence of the inflammatory cocktail with and without secretome were analysed by multiplex using the 48-Plex Bio-Plex Pro human cytokine screening panel (Bio-Rad Laboratories, USA) according to manufacturer’s instructions. Sample–antibody–bead complexes were re-suspended in sheath fluid for acquisition on the FLEXMAP® 3D (Luminex) using xPONENT® 4.0 (Luminex) software. Data analysis was done on Bio-Plex Manager™6.1.1 (Bio-Rad). Standard curves were generated with a five-parameter logistic (5-PL) algorithm, reporting values for both mean florescence intensity (MFI) and concentration data.

### Cell proliferation

Cell proliferation was assessed in 96-well tissue culture plates using non-immortalized human adult dermal fibroblasts (HDF) and HaCaT keratinocytes. Cells were seeded in culture medium (DMEM with 5% FBS, 1× GlutaMAX, 1× NEAA, 1× penicillin–streptomycin) with and without an inflammatory cocktail comprising similarly of IFNg, and/or PMA, and/or LPS. Secretome was included on a subset of cells seeded with the inflammatory cocktail. Culture medium only was used as a positive control for normal proliferation response. Cells were then incubated for 3 days at 37 °C in a CO_2_ incubator. After incubation, the cells were washed with HBSS before fixing in 1% PFA for 30 min at room temperature. After fixation, cells were stained with 0.5% crystal violet solution for 10 min at room temperature. The crystal violet was removed and the cells washed with water until the solution was clear. Cells were air-dried and 1N acetic acid solution was added to the cells to solubilise the crystal violet. After solubilisation, the absorbance was read at 560 nm using a spectrophotometer (Victor 3, 1420, Perkin Elmer, USA) and cells in each well were quantified by comparing the absorbance with the absorbance of a cell standard which was stained and solubilised using the same method.

### Equipment, reagents and chemicals

HDF was from Lonza, USA. HaCaT keratinocytes was from AddexBio, USA. DMEM, GlutaMAX, NEAA, TrypLE, PFA, and StemPro™ chondrogenesis/osteogenesis differentiation kit were from Gibco; Life Technologies, USA. FBS and HBSS were from HyClone; Cytiva, USA. Penicillin–streptomycin was from Biowest, France. PMA, LPS, and oil red O were from Sigma Aldrich, USA. Alizarin red and alcian blue were from Merck Millipore, Germany. Cryostor10 was from Biolife Solutions, USA. MesenCult™ adipogenic differentiation kit was from Stem Cell Technologies, Canada. Safranin O and crystal violet staining solutions were from Fisher Scientific, USA. PBS and acetic acid solution were from VWR; Avantor, USA. Human CD34, CD45, CD73, CD166, CD14 and CD90 antibodies, and IFNg, were from Miltenyi Biotech, Germany. Human CD105 antibody was from Biolegend, USA. The 6-well and 96-well tissue culture plates and HYPERFlasks® were from Corning, USA.

All images were acquired using an Olympus IX71 inverted microscope (Olympus, Japan).

### Statistical analysis

Real Statistics Resource Pack software (Release 8.8.1) for Microsoft Excel (Office 365) was used for data analyses. Data are expressed as mean ± standard deviation (SD). Comparison between groups were performed using one way ANOVA with Tukey’s post hoc test using a two-tailed test. *P* values less than 0.05 were considered statistically significant.

## Results

**Fig. 1:**
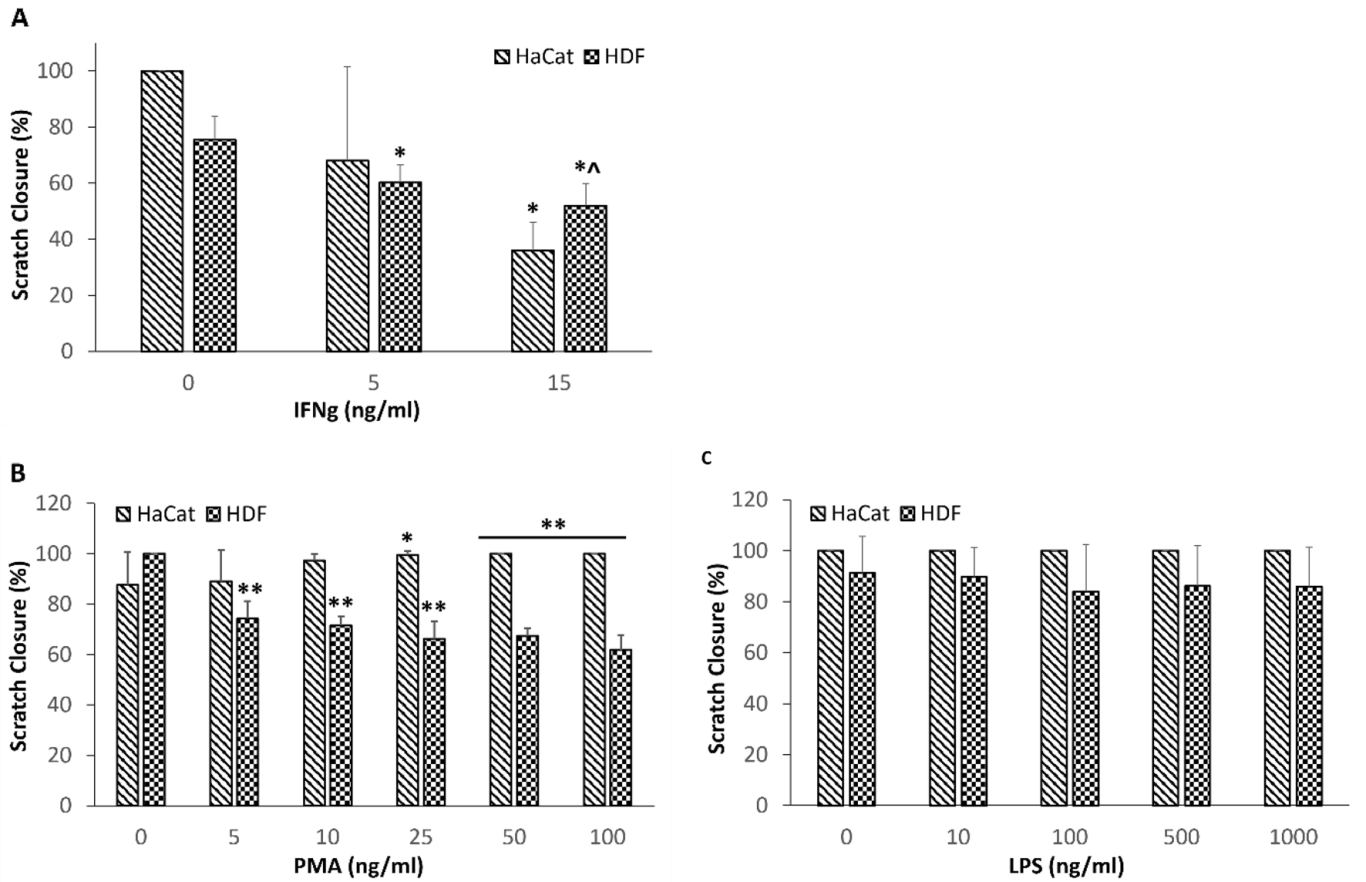
Effects of **a** IFNg, **b** PMA and **c** LPS on scratch closure in HaCaT and HDF cells after three and two days, respectively. Data (*N* = 6 biological replicates over two experiments) are expressed as mean ± standard deviaition (SD). *P < 0.05 versus 0 ng/ml IFNg, **P < 0.01 versus 0 ng/ml IFNg, ^*P* < 0.05 vs 5 ng/ml IFNg

We first assessed the ability of IFNg, PMA and LPS to independently inhibit scratch closures in both HaCaT and HDF. Scratch closure was assessed after incubation for three days for HaCaT cells and after 2 days for HDF cells. These timepoints were selected for each cell type as the scratch closure extent showed the greatest difference between cells that were treated with assay medium alone and those that were treated with assay medium containing inflammatory molecules. IFNg inhibited scratch closures in both cell types, with greater inhibition seen at a higher dose (Fig. 1a). PMA promoted HaCaT scratch closure while inhibiting that of HDF (Fig. 1b) while LPS did not significantly affect scratch closure in either cell type (Fig. 1c).

**Fig. 2:**
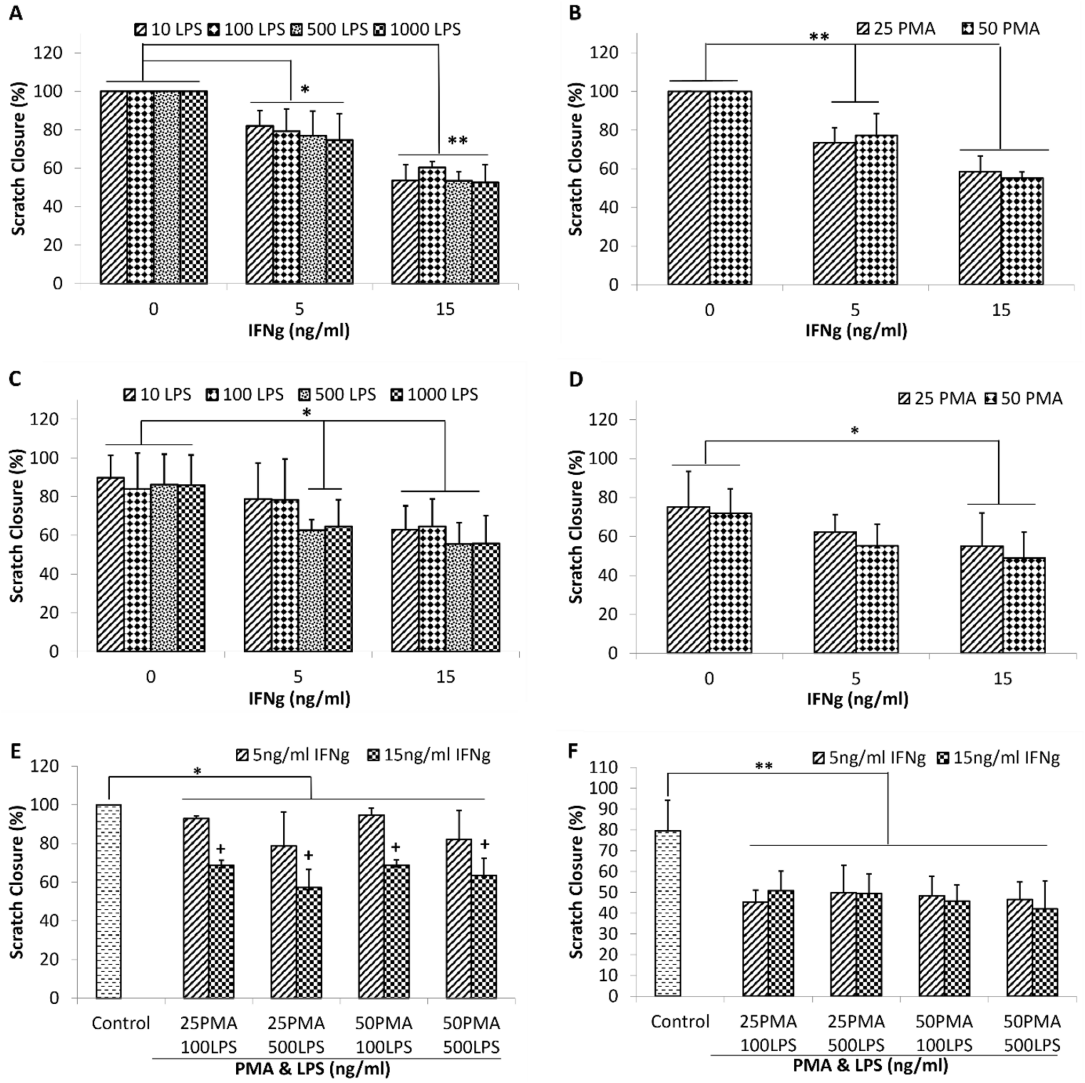
Effects of PMA and LPS and in combination on IFNg-induced inhibition of scratch closure in HaCaT (3-day incubation) and HDF (2-day incubation) cells. Graphs shows IFNg-induced inhibition of scratch closure in HaCaT cells by **a** 0–1000 ng/ml LPS and **b** 0–50 ng/ml PMA. Graphs show IFNg induced inhibition of scratch closure in HDF cells by **c** 0–1000 ng/ml LPS and **d** 0–50 ng/ml PMA. Graphs show different combinations of PMA and LPS on IFNg-induced inhibition of scratch closure in **e** HaCaT and **f** HDF cells. Control refers to assay diluent (culture medium). Data (*N* = 6–11 biological replicates over three experiments) are expressed as mean ± SD. *P < 0.05 versus 0 ng/mL IFNg or control, **P < 0.01 versus 0 ng/ml IFNg or control, ^**+**^*P* < 0.05 versus 5 ng/ml IFNg

Based on the results above, IFNg appeared to be a primary mediator inhibiting scratch closure in both cell types. We next evaluated the effect of PMA and LPS in combination with IFNg in the same setup. No dose–response effect of LPS or PMA treatment was observed for HaCaT scratch closure (Fig. 2a & b, respectively). In contrast, LPS at higher concentrations (500 and 1000 ng/mL) together with low dose IFNg (5 ng/mL) resulted in inhibition of HDF scratch closure similar to that of higher dose IFNg (15 ng/mL) with LPS (Fig. 2c). No dose–response effect of PMA with IFNg was observed for HDF scratch closure (Fig. 2d). Addition of all three molecules together resulted in consistent and significant scratch closure inhibition in both HaCaT and HDF cells (Fig. 2e and f, respectively) compared with the control. This was especially notable at the higher IFNg concentration (15 ng/mL) for HaCaT cells (Fig. 2e). Based on these findings, the combination of IFNg (15 ng/ml), PMA (25 ng/ml) and LPS (500 ng/ml) was selected as the “inflammatory cocktail” for both cell types to create consistent delayed scratch closures for subsequent experiments. This combination best mimicked the delayed healing observed in chronic wounds, and did not elicit any observable abnormal or unexpected change in the morphology of either cell type after addition of the factors.

**Fig. 3:**
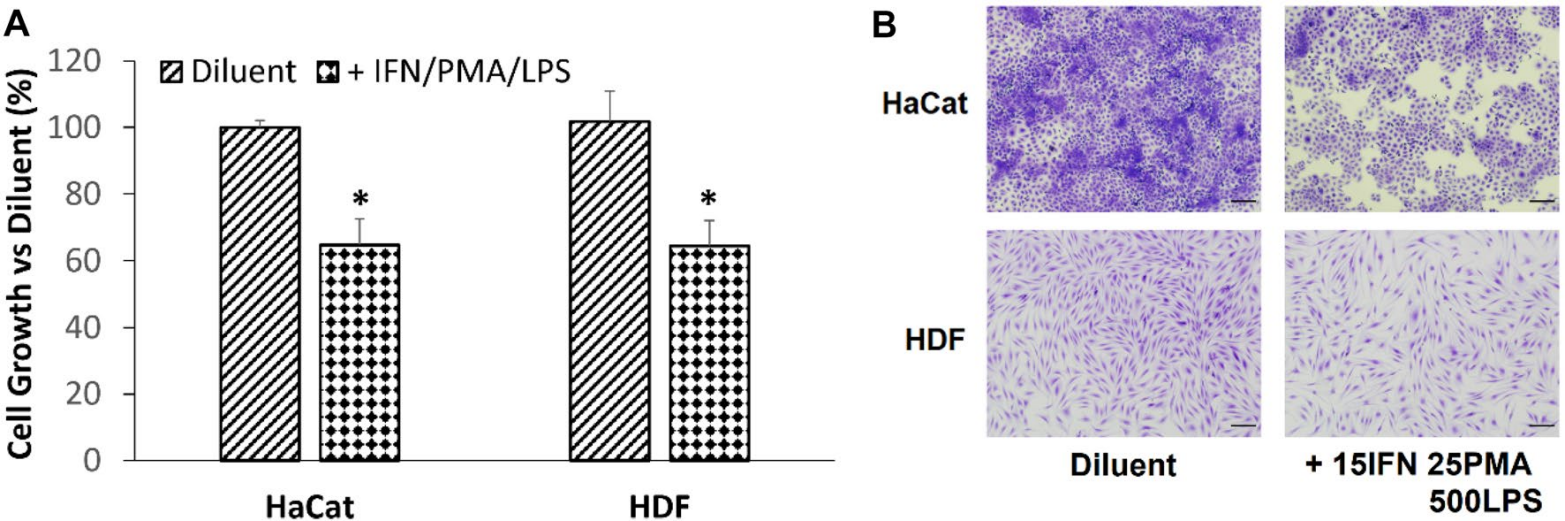
Effect of inflammatory cocktail on proliferation in HaCaT and HDF cells after 3 days. **a** Graph shows percentage cell proliferation compared with assay diluent (culture medium). **b** Microscopic images of HaCaT and HDF cells stained with crystal violet after proliferation assay. Data (*N* = 12 biological replicates over three experiments) are expressed as mean ± SD. *P < 0.01 versus diluent. Scale bar, 200 μm

Since wound closure involves both cell proliferation and migration, the effect of inflammatory cocktail on HaCaT and HDF proliferation was investigated. After a 3-day incubation period, the selected combination of IFNg, PMA and LPS significantly reduced cell proliferation in both HaCaT and HDF cells to a similar extent (Fig. 3), suggesting that the delay in scratch closure could be at least partly due to an inhibition of cell proliferation. Similarly, this timepoint was selected as it showed the greatest difference in cell numbers (indicative of proliferation rate) between cells that were treated with assay medium alone and those that were treated with assay medium containing the selected combination of IFNg, PMA and LPS.

The changes in cytokine levels in the cell culture elicited by the inflammatory cocktail were analysed using an antibody-based multiplex screening panel which included cytokines implicated in non-healing wounds. Altogether, 48 cytokines were measured in the culture supernatant of HDF and HaCaT cells before and after stimulation with the inflammatory cocktail, giving over 200 data points for comparison. To make this data set more digestible, a table to summarise either increases or decreases of each cytokine was generated (Table 1).

**Table 1:** Cytokines in HaCat and HDF culture medium after treatment with IFNg/PMA/LPS

Cytokine	Cytokine changes by IFNg/PMA/LPS		Cytokine	Cytokine changes by IFNg/PMA/LPS	
	HaCat	HDF		HaCat	HDF
Basic FGF	+	+	IL-4	NC	P
B-NGF	NC	NC	IL-5	P	+ +
CTACK	–	+ +	IL-6	+ +	+ +
Eotaxin	P	+ +	IL-7	–	NC
G-CSF	–	P	IL-8	–	+
GM-CSF	NC	P	IL-9	+	+ +
GRO-a	–	+ +	IP-10	+ +	+ +
HGF	NC	+	LIF	NC	+ +
IFN-a2	NC	P	MCP-1	NC	NC
IFNg	NA	NA	MCP-3	NC	+ +
IL-10	–	P	M-CSF	+	NC
IL-12(p40)	NC	P	MIF	+	NC
IL-12(p70)	NC	P	MIG	+ +	P
IL-13	NC	P	MIP-1a	+	P
IL-15	NC	P	MIP-1b	NC	+ +
IL-16	+	P	PDGF-BB	NC	P
IL-17	NC	+ +	RANTES	NC	+ +
IL-18	NC	+	SCF	–	NC
IL-1a	+	+ +	SCGF-b	NC	NC
IL-1b	+ +	P	SDF-1a	NC	NC
IL-1ra	NC	P	TNF-a	NC	P
IL-2	P	P	TNF-b	+	P
IL-2Ra	+	+ +	TRAIL	–	P
IL-3	NC	P	VEGF	NC	P

Changes in analysed cytokines are in comparison with treatment with culture medium alone. + , two to fivefold increase; + + , greater than fivefold increase; −, two to fivefold decrease; – –, greater than fivefold decrease; *P* present after inflammatory cocktail treatment while absent after culture medium alone, *NC* no significant change (less than twofold increase or decrease). Data are from 12 biological replicates pooled from three experiments and analysed in duplicates (two technical repeats)

The majority of inflammatory cytokines measured were induced in HDF following treatment with the inflammatory cocktail. Some that were undetectable under normal conditions were detected after treatment with the inflammatory cocktail. Importantly, these include cytokines such as tumour necrosis factor alpha (TNFa) and interleukin 1 beta (IL-1b) that are also upregulated in chronic wounds. Many induced cytokines had a greater than fivefold increase (Table 1), of which GROα, IL-6, IL-17 and IP-10, etc. are notable. In HaCaT cells, a much smaller number of inflammatory cytokines were induced following similar treatment (16 versus 39 in HDF). IL-1b, IL-6, IP-10 and monokine induced by gamma (MIG) showed a fivefold or greater increase, while cytokines including granulocyte colony stimulating factor (G-CSF), IL-10 and TNF-related apoptosis-inducing ligand (TRAIL) showed reduced levels. Thus, through the use of titrated amounts of IFNg, LPS and PMA, we demonstrated the ability of this in vitro assay system to have delayed closure and show expression of pro-inflammatory markers, majority of which are present in chronic wounds in vivo.

**Fig. 4:**
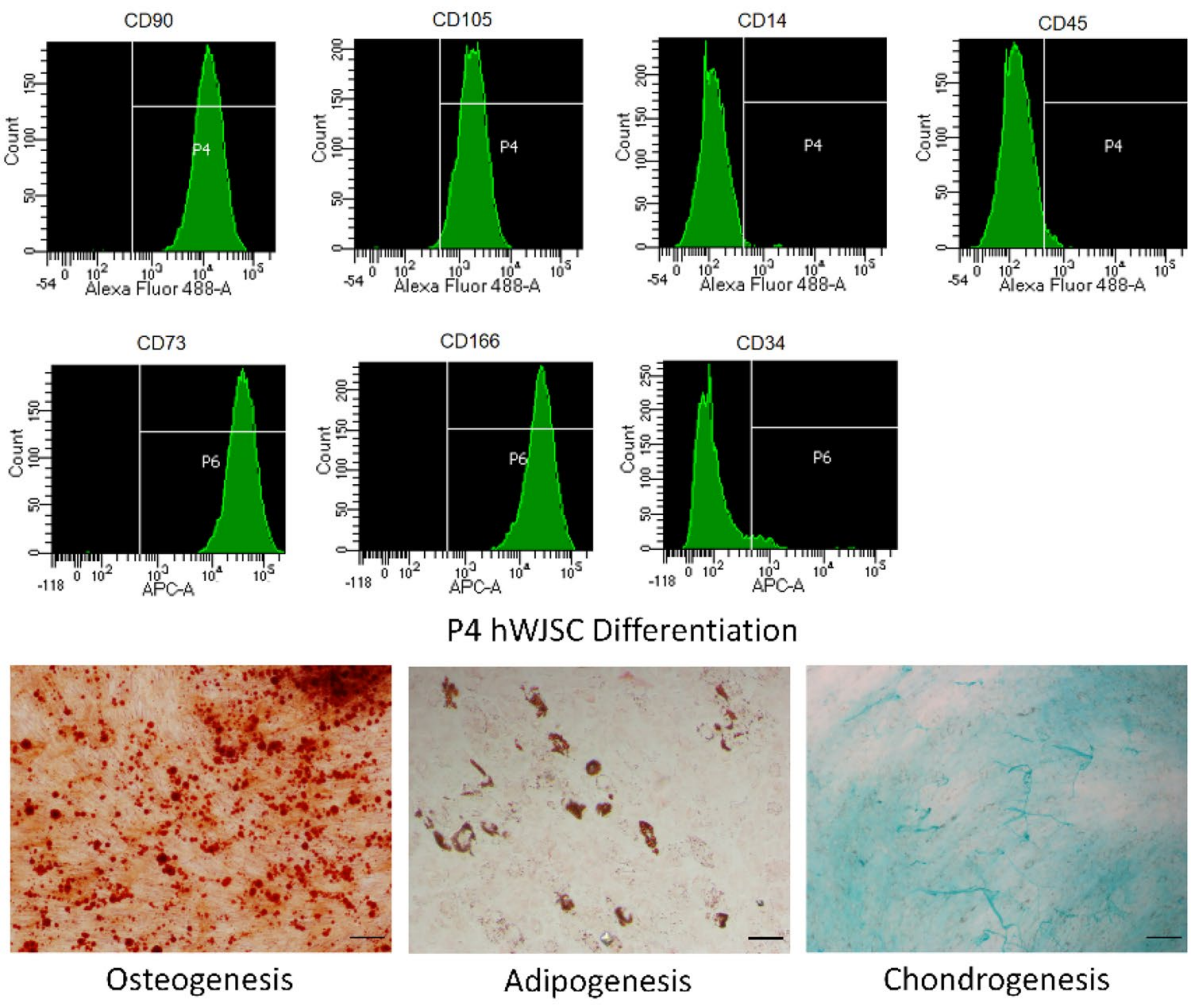
hWJSC characterisation. Top panel shows cell surface marker expression of hWJSCs at passage 3 by flow cytometry, with cells on the right and left side of the gate showing positive and negative marker expression, respectively. Bottom panel shows differentiation capacity of hWJSCs at passage 4. Reddish-brown deposits indicate positive osteogenesis, orange-brown droplets indicate positive adipogenesis, intense blue extracellular streaks indicate positive chondrogenesis. Representative data shown from four biological replicates over two experiments. Scale bar, 200 μm

Given that the assay system displayed some features of chronic wounds, i.e. delayed scratch closure and induced expression of inflammatory cytokines, we sought to test the effect of a proprietary secretome product developed in-house for healing chronic wounds using this system. The secretome was derived from human umbilical Wharton’s jelly stem cells (hWJSCs) which were mesenchymal-like and exhibit the requisite cell surface markers and trilineage differentiation capability characteristic of these cells (Fig. 4).

**Fig. 5:**
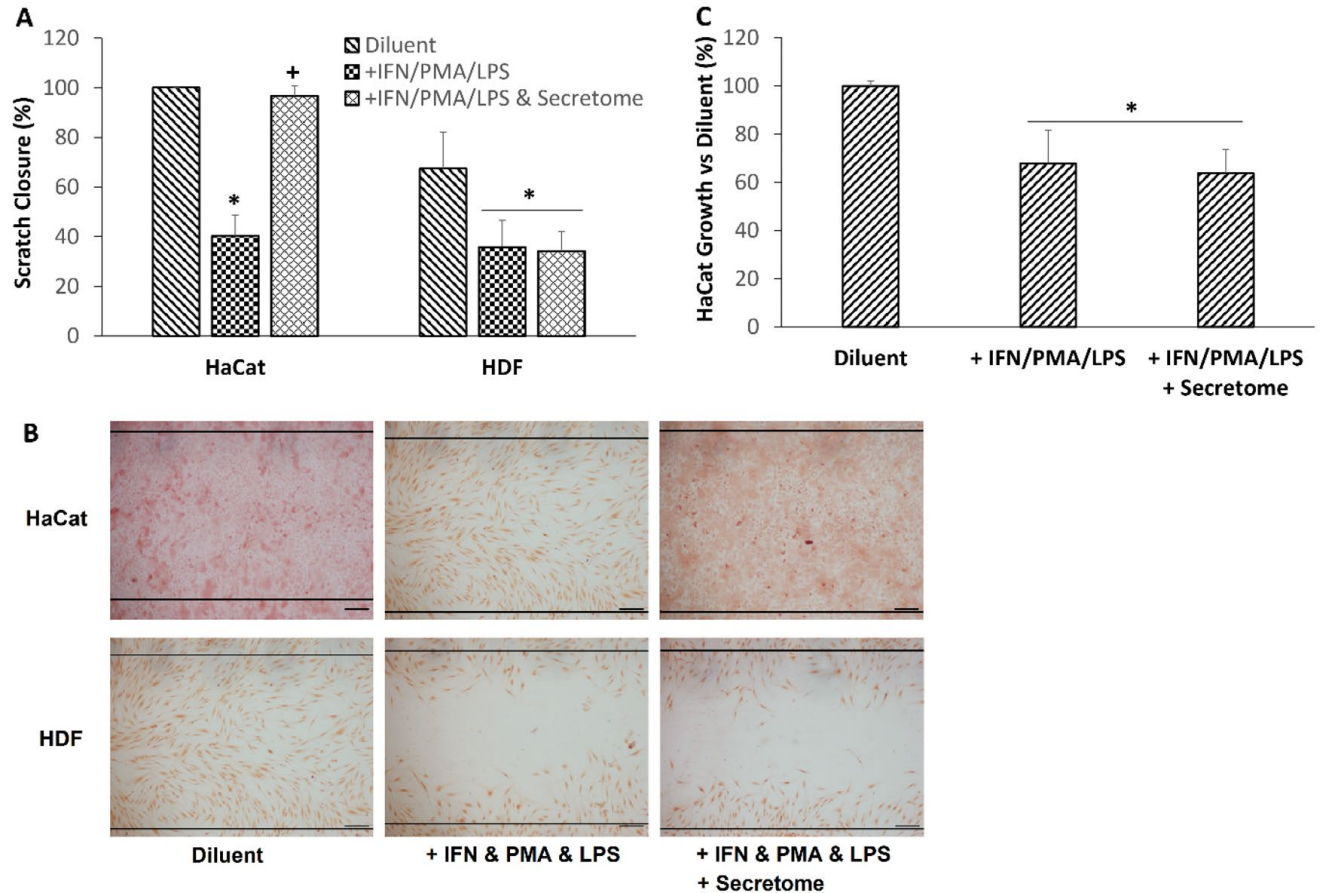
Effects of secretome on scratch closure in HaCaT (3-day incubation) and HDF (2-day incubation) cells after treatment with inflammatory cocktail with and without hWJSCs secretome. **a** Graph shows percentage scratch closure in HaCaT and HDF cells treated with inflammatory cocktail with and without hWJSCs secretome. **b** Microscopic image of cells in the scratch zone (between the two black lines) after treatment with inflammatory cocktail with and without hWJSCs secretome. Scale bar , 200 μm. **c** Graph shows proliferation in HaCaT cells (three day incubation) treated with inflammatory cocktail with and without secretome. Diluent refers to assay diluent (culture medium). Data (*N* = 12 biological replicates over three experiments) are expressed as mean ± SD. *P < 0.01 versus diluent, ^**+**^*P* < 0.01 versus without secretome

This secretome has been shown to strongly promote acute wound healing in vitro and in vivo (WO 2023/101603 A1, manuscript in preparation). Treatment with the secretome together with the inflammatory cocktail led to scratch closure in HaCaT cells after the three day incubation period but had no effect on similarly treated HDF cells after the two day incubation period (Fig. 5a & b). Assessment of changes in cytokine levels upon secretome treatment were not performed as many of the analysed factors produced by the HaCaT and HDF cells in our model shown in Table 1 were also present in the secretome (data not shown). This made it difficult to determine the effect of the secretome on the levels of these factors secreted by the cells used in this model. Since the combination of IFNg, PMA and LPS was shown to inhibit cell proliferation, we then sought to determine if the restoration of HaCaT scratch closure ability by the secretome was due in part to reversal of the inhibited cell proliferation. Surprisingly, addition of our secretome had no effect on the reduced proliferation induced by the inflammatory cocktail after the three day incubation period (Fig. 5c).

## Discussion

Treatments for chronic wounds largely focus on wound debridement methods such as surgery and/or use of medical grade maggots. Since these methods are performed as an outpatient procedure, they require the patient to travel to medical centres and receive input from doctors or nurses. There is a clear need for novel therapeutics for chronic wounds which can be administered by the patients themselves at home. To accelerate the discovery of such therapeutics, a reliable in vitro assay that can accurately model a chronic wound and that is able to be used in a high-throughput format for screening would be valuable. Currently, commonly used models of chronic inflammation (Erol et al. 2018; Wiegand et al. 2024) often utilise direct addition of pro-inflammatory cytokines, such as tumour necrosis factor alpha and interleukin 1 beta, to cells. However, the use of a few pro-inflammatory cytokines may not appropriately stimulate the complex response as compared with when they are exposed to pro-inflammatory substances (e.g. LPS, PMA) that would elicit a more physiological inflammation response including the production of these cytokines. A recent review has summarized the commonly available wound healing models reported and has discussed their advantages and issues, in consideration of their relevance and translational potential to humans (Flynn et al. 2023).

When developing the assay, careful thought was given as to which molecules would be best to stimulate and then mimic the chronic wound microenvironment in a culture dish. IFNg was chosen as it is a crucial cytokine involved in the immunity of wound healing. However, understanding its role and effect on wound healing and on chronic wounds has been complex as both low and high levels of IFNg have been associated with poor wound healing. Low levels of IFNg have been observed to be associated with impaired wound closure in mice (Kanno et al. 2019) and non-healing DFUs (Theocharidis et al. 2020) while high levels of IFNg have been observed in the wound fluid of some chronic inflammatory skin conditions (Banerjee et al. 2017) and have been shown to inhibit wound healing in both in vitro and in vivo models (Laato et al. 2001). In addition, the effects of IFNg observed in these studies are also mediated by its influence on immune cell infiltration and consequent inflammation (Schroder et al. 2004), which cannot be replicated in a 2D cell culture model. Due to the complex role of IFNg in the chronic wound microenvironment, we sought to test different doses of IFNg in developing this assay system. While both doses tested in the assay resulted in a delay in scratch closure, the higher dose of 15 ng/mL resulted in a more consistent and greater delay in both HaCaT and HDF (Fig. 1A) and this is considered to be more relevant to chronic wounds.

PMA is a low-cost molecule and has been well established in previous studies to induce chronic inflammation (Waskow et al. 2007; Muller et al. 2008). PMA is frequently used to differentiate monocytes to macrophages in inflammation studies and is needed to maintain the cells in a differentiated state and would be useful to include for future development of a co-culture model with macrophages to provide an immune component. While it is known to activate protein kinase C and play a role in several wound healing processes such as cell signalling, angiogenesis and ECM remodelling, it is also known to cause skin irritation and be a potent inducer of inflammation. In fact, PMA is a potent stimulator of M1-type inflammation (Marinescu et al. 2022), and chronic wounds are known to have persistent M1-type inflammation (Krzyszczyk et al. 2018). The contradictory effect of PMA on wound healing was observed in his study as it was seen that PMA promoted HaCaT scratch closure while inhibiting the same in HDF cells (Fig. 1b). It is not known if these opposing outcomes resulted from the same or different cellular pathways but it seems likely that stimulation of immune pathways was responsible in both cases as inflammatory cytokines were induced; therefore, both can still be equally relevant to wound healing processes.

LPS is a component of the bacterial membrane and known for its potent pro-inflammatory properties. It is another key molecule found to be significant in chronic wounds as exogenous bacterial infections causing biofilms is a major factor driving wounds to become chronic (Trøstrup et al. 2016). Although LPS alone did not significantly affect HaCaT or HDF scratch closure (Fig. 1c), probably due to lack of immune cells in this simplified chronic wound assay system, it did cause consistent and significant scratch closure inhibition in both cell types when in combination with IFNg and PMA (Fig. 2a & c). This is possibly due to the combination of factors triggering innate responses that represent chronic wounds at a fundamental level.

While it is known that inflammatory cytokines can cause morphological changes (Banno et al. 2004), there were no obvious changes in our cell types. This may be because our model is based on triggering physiologically relevant downstream inflammation responses, rather than directly using inflammatory cytokines. Importantly, this assay system utilising IFNg, PMA and LPS stimulation exhibited significant delayed scratch (wound) closure in HDF and HaCaT cells, which is a main feature associated with chronic wounds. Furthermore, it has been shown that dermal fibroblasts isolated from chronic wounds show a marked reduced propensity for proliferation and migration (Berberich et al. 2020; Monika et al. 2022). In these studies, chronic wound fibroblasts show an increased abundance of adhesion complexes, such as integrins, and reduced amounts of vimentin, an intermediate filament that enhances cell migration. In parallel, chronic wound fibroblasts also show an impaired ability to proliferate due to down regulation of DNA replication factors. Consequently, chronically inflamed fibroblasts show very poor migration. In Berberich’s study, a week-long combinatorial pre-treatment of chronic wound fibroblasts in a non-inflammatory environment with ruxolitinib (inhibits pro-inflammatory signalling) and 5-azacytidine (inhibits DNA methylation) could only partially reverse the chronic wound phenotype and only for cell proliferation. Their data suggests that the migratory dysfunction in chronically inflamed fibroblasts is extremely hard to reverse and this may explain why in our model, the test substance (purified hWJSCs secretome) which strongly accelerates wound healing (WO 2023/101603 A1, manuscript in preparation) had little effect on scratch closure in HDFs treated with the inflammatory cocktail. In fact, our result provides further support that our model more closely resembles a chronic wound.

In addition to delayed scratch closure, the inflammatory cocktail also elicited changes in cytokine levels secreted by the cells, some of which are relevant to chronic wounds. The inflammatory cocktail treatment was able to induce TNFa in HDF and IL-1 in both HaCaT and HDF. This agrees with previous studies showing that HDF isolated from chronic wounds secrete moderately higher levels (~ 1.4 fold increase) of TNFa compared with acute wound fibroblasts (Monika et al. 2023) and increased levels of TNFa and IL1 were observed in chronic wounds (Barrientos et al. 2008). IL-17, which was elevated in cell culture supernatant from stimulated HDFs, has also been implicated in chronic inflammatory skin conditions and delayed wound healing (Hadian et al. 2019). A recent study examining elderly patient with non-healing wounds showed that these patients had relatively higher levels of cytokines including IL-4, IL-6, IL-8, FGF2, MCP1 and MIP1 compared with those with healing wounds (Krzystek-Korpacka et al. 2019). These cytokines, except for MCP1, were also increased in our assay system (HDF and/or HaCaT cells).

Taken together, the assay system we have developed clearly possesses some of the features of chronic wounds. Pivotally, we showcase its applicative potential by successfully promoting re-epithelialisation (scratch closure by HaCaT cells) using a novel, patent pending secretome derived from human Wharton’s jelly stem cells (hWJSCs) that has been shown to promote wound healing (WO 2023/101603 A1), highlighting its translational value for screening or testing potential therapeutic interventions. This demonstrates functionality of an easily accessible and affordable high-throughput cell culture assay system which allows for the examination of cellular behaviours, molecular pathways and therapeutic interventions relevant to chronic wound healing. However, it is important to note that it does not fully capture the intricate dynamics of an actual wound, as it lacks the cellular interplay between different cell types. There is also no immune cell involvement and angiogenesis that are crucial to wound healing as well as exogenous factors such as dirt and microorganisms. Nevertheless, we believe that our model would be more relevant for screening compounds and studying delayed wound healing compared with traditional scratch wound assays that more closely resemble acute wounds. Overall, the development of a more physiologically relevant in vitro wound healing model is a significant step towards gaining a comprehensive understanding of chronic wound healing mechanisms and advancing the field of wound care.

## Data Availability

The data that support the findings of this study are available from the corresponding author, P.M., upon reasonable request.
